# Roles of endothelial A-type lamins in migration of T cells on and under endothelial layers

**DOI:** 10.1038/srep23412

**Published:** 2016-03-21

**Authors:** Kwang Hoon Song, Jaehyun Lee, HyoungJun Park, Hye Mi Kim, Jeehun Park, Keon Woo Kwon, Junsang Doh

**Affiliations:** 1 Department of Mechanical Engineering, Pohang University of Science and Technology (POSTECH) San 31, Hyoja-dong, Nam-Gu, Pohang, Gyeongbuk, 790-784, Korea; 2 School of Interdisciplinary Bioscience and Bioengineering (I-Bio), Pohang University of Science and Technology (POSTECH) San 31, Hyoja-dong, Nam-Gu, Pohang, Gyeongbuk, 790-784, Korea; 3 Division of Integrative Bioscience and Biotechnology (IBB), Pohang University of Science and Technology (POSTECH) San 31, Hyoja-dong, Nam-Gu, Pohang, Gyeongbuk, 790-784, Korea

## Abstract

Stiff nuclei in cell-dense microenvironments may serve as distinct biomechanical cues for cell migration, but such a possibility has not been tested experimentally. As a first step addressing this question, we altered nuclear stiffness of endothelial cells (ECs) by reducing the expression of A-type lamins using siRNA, and investigated the migration of T cells on and under EC layers. While most T cells crawling on control EC layers avoided crossing over EC nuclei, a significantly higher fraction of T cells on EC layers with reduced expression of A-type lamins crossed over EC nuclei. This result suggests that stiff EC nuclei underlying T cells may serve as “duro-repulsive” cues to direct T cell migration toward less stiff EC cytoplasm. During subendothelial migration under EC layers with reduced expression of A-type lamins, T cells made prolonged contact and substantially deformed EC nuclei, resulting in reduced speed and directional persistence. This result suggests that EC nuclear stiffness promotes fast and directionally persistent subendothelial migration of T cells by allowing minimum interaction between T cells and EC nuclei.

Lamins are intermediate filaments that form the supportive meshwork underlying the inner nuclear membrane of eukaryotic cells. There are two types of lamins in most mammalian cells, A-type lamins (lamin A and C) and B-type lamins (lamin B1 and B2), and both contribute to various cellular functions as well as nucleus mechanics^1^,^2^,^3^,^4^,^5^. Expression levels of A-type lamin, or the ratio between A-type lamin and B-type lamin, determine nuclear stiffness^6^,^7^,^8^.

Cancer cells and leukocytes often migrate through narrow spaces such as blood vessels and dense 3D interstitial spaces^9^,^10^. Because stiffness of nucleus is an order of magnitude higher than that of cytoplasm^11^,^12^,^13^, nuclear stiffness determined by the expression levels of A-type lamins has shown to be a major hurdle of cell migration in confined microenvironments. For example, neutrophils known to express low levels of lamin A can pass through narrow pores, while neutrophils overexpressing lamin A lack such capability^14^; partial knockdown of A-type lamins in cancer cells significantly increased 3D migration speed, while overexpression of A-type lamins reduced 3D migration speed^15^. Most experiments mimicking confined microenvironments have been performed in acellular systems, such as collagen matrixes^16^,^17^, porous membranes^14^,^15^, and microchannels^18^,^19^, but confined microenvironments *in vivo* are composed of layers and networks of cells as well as a meshwork of fibrillar extracellular matrixes. Therefore, the nuclear stiffness of cells comprising confined microenvironments may serve as distinct biomechanical cues or physical barriers for the migration of invading cancer cells or leukocytes.

T cells are highly motile cells responsible for antigen-specific cell-mediated immune responses^20^,^21^. T cells in the blood stream infiltrate tissues to perform immune responses. For tissue infiltration, T cells undergo a series of leukocyte adhesion cascade, rolling, firm adhesion, intraluminal crawling, and transendothelial migration (TEM) (Fig. 1), to breach endothelium^22^,^23^. There is ample evidence that T cells interact with the stiff nuclei of underlying endothelial cells (ECs) during intraluminal crawling by generating cdc42-dependent F-actin-rich tips, invasive filopodia^24^ or invadosome like protrusions (ILPs)^25^,^26^,^27^. Dynamic imaging has revealed that ILPs formed in T cells probed underlying ECs and significantly deformed EC nuclear lamina to find spots for TEM with minimal resistance^27^. We also observed that crawling T cells avoid crossing over EC nuclei^28^. Considering that cdc42-inhibited T cells frequently cross over EC nuclei, it is likely that T cells sense underlying EC nuclei by cdc42-dependent invasive F-actin-rich tips to steer the crawling direction and optimize intraluminal crawling pathway. However, the role of nuclear stiffness on intraluminal crawling and subsequent TEM has not been investigated. After TEM, leukocytes underneath the endothelium migrate substantial distances to breach the basement membrane and reach interstitial spaces^22^,^29^ (Fig. 1). During this subendothelial migration, leukocytes migrating in the narrow gaps between the layers of ECs and pericytes/basement membranes are likely to interact with EC nuclei. Likewise, migration of leukocytes in tissues densely packed with cells, such as lymph nodes and spleen, would be affected by the stiff nuclei of other cells. However, the effects of nuclear stiffness of cells surrounding leukocytes on migration have not been elucidated.

To address how EC nuclear stiffness affects the migration of T cells on and under EC layers, we reduced expression levels of A-type lamins in ECs using a small interfering RNA (siRNA) targeting the gene encoding A-type lamin (LMNA) to decrease nuclear stiffness. Then, the motility patterns of T cells interacting with LMNA knockdown (LMNA-KD) ECs were compared with those of T cells interacting with control siRNA-treated ECs (control ECs) or untreated ECs. T cells on LMNA-KD ECs crossed over EC nuclei more frequently than T cells on control or untreated ECs, indicating stiff nuclei of ECs can serve as “duro-repulsive” cues for guiding T cells crawling on EC layers. T cells under LMNA-KD ECs exhibited slower and less persistent migration because of prolonged interactions with EC nuclei that significantly deformed these EC nuclei compared with T cells under control or untreated ECs. These results suggest that stiff EC nuclei can promote fast and directionally persistent migration by allowing relatively minimal interactions between EC nuclei and T cells underneath.

## Results

### Characterization of EC LMNA-KD by siRNA transfection

**Figure 2:**
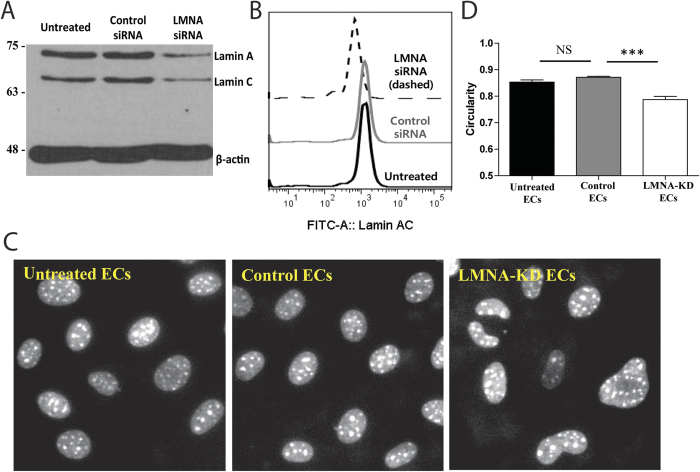
Characterization of EC LMNA knock down by siRNA transfection. (**A**,**B**) Western blot (**A**) and Flow cytometry (**B**) analysis showing expression levels of A-type lamins (lamin **A** and **C**) in untreated, control and LMNA-KD ECs. β-actin antibody (bottom) was used as a loading control. (**C**) Fluorescence images of nuclei of untreated, control and LMNA-KD ECs (stained with DAPI). (**D**) Quantification of nuclear circularity of untreated, control and LMNA-KD ECs. Data are representative of 5 independent experiments, (*n* = 50 for each condition) (Mann-Whitney U-test, two-tailed, NS: not significant, ***p < 0.001).

In order to study the influence of EC A-type lamins (or EC nuclear stiffness) on the motility of T cells, the amount of A-type lamins in ECs was reduced by transfection of an siRNA targeting LMNA. As shown in Fig. 2A,B, the expression level of A-type lamins in ECs treated with LMNA siRNA (LMNA-KD ECs) was significantly lower than that in untreated ECs. Typically, ~50% reduction in the expression of A-type lamin was achieved. A-type lamin expression was not affected by control siRNA treatment. Similar to previous reports^8^,^30^, reduced expression of A-type lamins caused significant morphological changes in EC nuclei potentially due to decreased stiffness of the nuclei; the nuclei of LMNA-KD ECs were significantly distorted, while the nuclei of untreated or control siRNA-treated ECs (control ECs) were smooth and elliptical (Fig. 2C). To quantitatively assess the alteration in nuclear morphology resulting from reduced expression of A-type lamins, nuclear circularity (4π × area of nucleus/perimeter^2^) of ECs^31^ in each experimental condition was calculated and plotted (Fig. 2D). The nuclear circularity of LMNA-KD ECs was significantly lower than that of untreated and control ECs, confirming that the reduced expression of A-type lamins significantly perturbed EC nuclear morphology presumably by softening the supportive network underlying the nuclear envelope.

**Figure 3:**
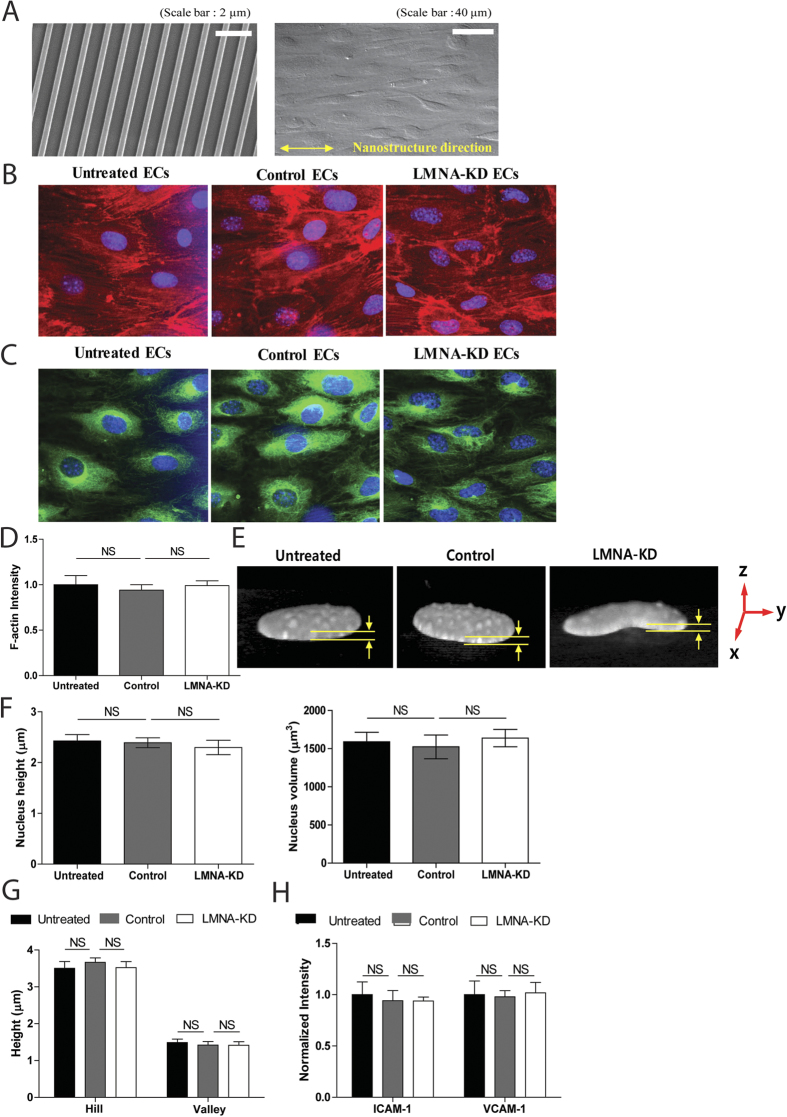
Effect of reduced expression of A-type lamins on cytoskeletal structure, topography, and adhesion molecule expression in EC layers. (**A**) Representative scanning electron microscope (SEM) image of nanostructured (350 nm/700 nm/300 nm, ridge/groove/depth) surfaces to align ECs and differential interference contrast (DIC) image of well-aligned EC layer formed on the nanostructured surface. (**B**,**C**) Confocal fluorescence images of F-actin (**B**) and microtubule (**C**) overlaid with nucleus (blue). (**D**) Average fluorescence intensities of F-actin, (*n* = 4 for each condition). (**E**,**F**) 3D-reconstructed images of nucleus (**E**) and nuclear height and volume (**F**) measured from 3D reconstruction of z-section images, (*n* = 10 for each condition). (**G**) Heights of hills and valleys of 3D reconstructed images, (*n* = 10 for each condition). (**H**) Expression levels of ICAM-1 and VCAM-1 on ECs measured by immunofluorescence microscopy, (ICAM-1, *n* = 5; VCAM-1, *n* = 6).

Nuclear lamins regulate gene expression and link the nucleus and cytoskeleton^1^,^6^,^32^,^33^, thus LMNA-KD may alter EC cytoskeleton structure, EC layer topography, and/or molecular expressions patterns in addition to nucleus stiffness and shape. Such alterations in ECs can influence T cell-EC interactions; the EC phenotype, therefore, needs to be carefully characterized prior to the T cell migration study. Well-aligned confluent EC layers cultured on nanostructured surfaces (Fig. 3A), which mimic the alignment of ECs in native blood vessels *in vivo*^34^, were stimulated with TNF-α and characterized. First, cell density and cell alignment along the groove direction were measured (Fig. S1 in Supplementary Information (SI)). Neither cell density nor alignment of EC layers was significantly altered by LMNA-KD. Then, more detailed analyses of the cytoskeletal distribution and topography of EC layers were performed by confocal imaging of nuclei, F-actin, and microtubules. F-actin and microtubule distributions in EC layers appeared indistinguishable (Fig. 3B,C), and the fluorescence intensity of F-actin did not differ significantly among untreated, control, and LMNA-KD ECs (Fig. 3D), indicating that ~50% reduction in the expression of A-type lamins minimally perturbed nucleo-cytoskeletal coupling. Nuclear morphology analyzed by 3D reconstruction of z-section images revealed that LMNA-KD significantly altered the nuclear shape in the xy-plane (as shown in Fig. 2B) without significantly affecting the nuclear height and volume (Fig. 3E,F). In addition, the overall topography of LMNA-KD EC layers, as assessed by measuring the height of the hills and valleys in 3D reconstructed topographical landscapes (detailed measurement method is described in Fig. S2 of SI), was not significantly different from that of control or untreated EC layers (Fig. 3G). Finally, the expression levels of adhesion molecules such as intercellular adhesion molecule 1 (ICAM-1) and vascular adhesion molecule 1 (VCAM-1) on ECs, as measured by fluorescence microscopy, were comparable among control, untreated, and LMNA-KD EC layers (Fig. 3H), indicating that ICAM-1 and VCAM-1 expression levels on EC surfaces were not affected by LMNA-KD. Taken together, ~50% reduction in the expression of A-type lamins by siRNA treatment minimally affected the cytoskeletal structure, topography, and adhesion molecule expression in ECs that can potentially influence migration of T cells.

### Experimental settings to assess roles of endothelial A-type lamins in T cell migration on and under EC layers

**Figure 4:**
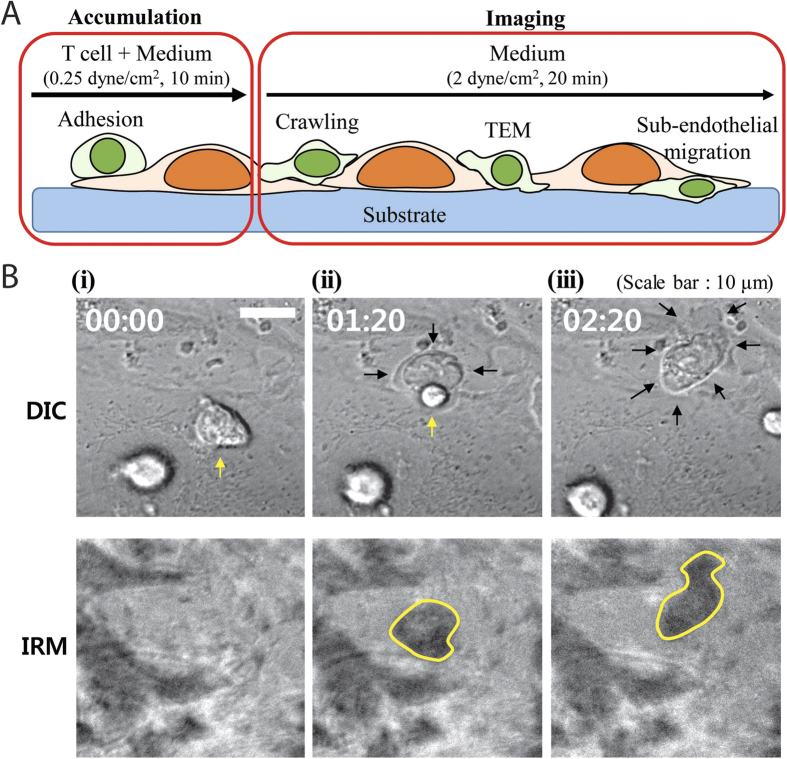
Experimental settings to monitor T cell migration on and under EC layers. (**A**) Schematic illustration of experimental procedures and T cell dynamics. (**B**) Time-lapse differential interference contrast (DIC) images (upper) and interference reflection microscopy (IRM) images (lower) of T cells migrating on EC layers (i), undergoing TEM (ii) and migrating under EC layers (iii).

With these effects of LMNA-KD by siRNA, we investigated roles of EC A-type lamins in regulating motility of T cells. DO11.10 T cell blasts, *in vitro* activated murine primary CD4+T cells, were used. *In vitro* activated T cells have been widely used for cancer immunotherapy by intravenous injection^35^,^36^, indicating they have excellent extravasation characteristics *in vivo*. As schematically shown in Fig. 4A, parallel flow chamber assays were performed using monolayers of ECs stimulated with TNF-α. Well-aligned confluent EC layers cultured on nanostructured surfaces with flow applied to the direction of EC orientation were used for the bulk of the experiments. To maximize number of T cells within an imaging field of view, T cells were first accumulated on ECs by perfusing suspension of T cells in culture medium at low shear stress (0.25 dynes/cm^2^) for 10 min. Then, the blank culture medium at the elevated shear stress (2 dynes/cm^2^), a typical shear stress used *in vitro* assays for leukocyte adhesions^37^,^38^, was applied to the T cells crawling on ECs and time-lapse images with 15 s-interval were acquired for the next 20 min. One of the most critical factors in this experiment is distinguishing T cells on EC layers and T cells under EC layers, which are separated in z-direction by the EC layers with ~5-μm thickness. We thought we could harness DIC imaging which generates dark shadows for slightly out-of-focus objects^39^,^40^,^41^. If we focus EC layers and apply T cells on the EC layers, the T cells on the EC layers would be located slightly out of the focal plane with dark shadows surrounding the T cells in DIC images. By performing transendothelial migration (TEM), the T cells on the EC layers, or located slightly over the focal plane, would move underneath the EC layers, or move to the focal plane, resulting in disappearance of dark shadows surrounding the T cells. To further confirm that T cells on EC layers and T cells under EC layers can be distinguished by the presence or absence of dark shadows in DIC images, interference reflection microscopy (IRM) was performed along with DIC imaging. Objects within ~100 nm generate signal for IRM images^42^,^43^, thus only T cells under EC layers would generate dark spots and T cells on EC layers would not be visible in IRM images. Time-lapse DIC and IRM images of T cells undergoing TEM are shown in Fig. 4B and Movie S1 in SI. As shown in Fig. 4B(i), a T cell with a dark shadow in a DIC image did not generate noticeable signals in an IRM image. In contrast, a T cell lacking a dark shadow in a DIC image generated dark spots in an IRM image (Fig. 4B(iii)). Importantly, a T cell undergoing TEM had dark shadow on one side in a DIC image and dark spots on the other side in an IRM image (Fig. 4B(ii)). These results mean that entire processes of TEM including intraluminal crawling, TEM, and subendothelial migration can be monitored and clearly distinguished by DIC imaging.

### Roles of endothelial A-type lamins on T cell migration on EC layers

**Figure 5:**
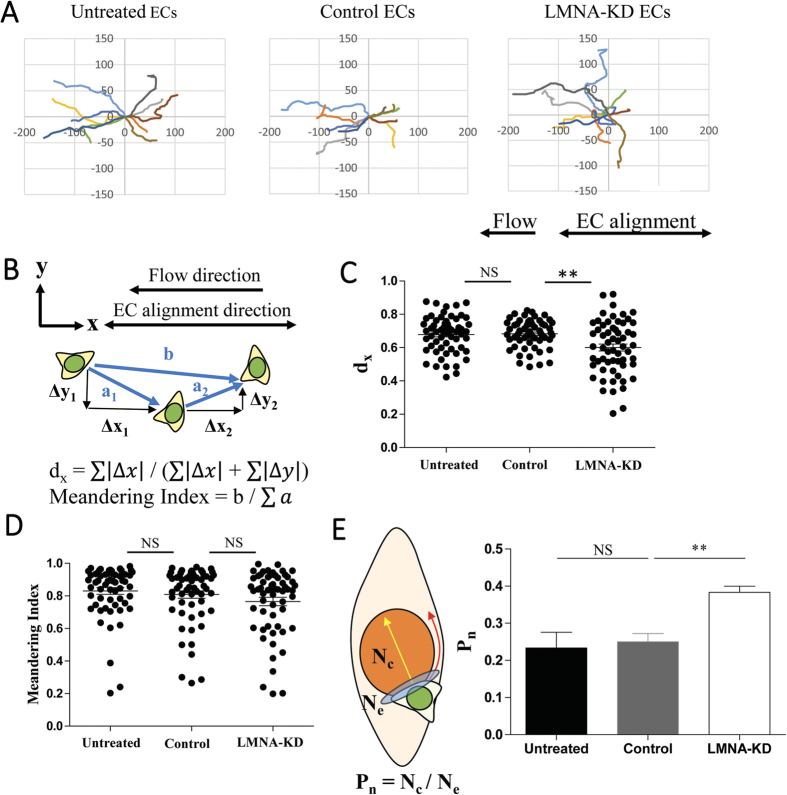
Effect of EC A-type lamins on T cell migration on EC layers. (**A**) Representative trajectories of crawling T cells on untreated, control and LMNA-KD ECs. (**B**) Definitions of parameters for quantitative T cell migration analysis. (**C**,**D**) Effect of A-type lamins on d_x_ values (**C**) and meandering indexes (**D**) of crawling T cells on untreated, control and LMNA-KD ECs. *n* = 60 for each condition. (**E**) Definition of probability to cross over nuclei of ECs (P_n_), and P_n_ values of T cells on untreated, control and LMNA-KD ECs. Untreated ECs, *n* = 151; Control ECs, *n* = 145; LMNA-KD ECs, *n* = 150 in total. Data are representative of 6 independent experiments (Mann-Whitney U-test, two-tailed, NS: not significant, **p < 0.001).

Representative trajectories of crawling T cells on EC layers with three different conditions, untreated, control, and LMNA-KD, were plotted in Fig. 5A. As previously reported, T cells on untreated and control EC layers crawled along the direction of EC orientation^28^. In contrast, T cells on LMNA-KD ECs appeared to crawl randomly compared with T cells on untreated or control ECs. Motility patterns of T cells on ECs in three different conditions were quantitatively analyzed by calculating directionality index (d_x_)^44^, which measures directionality of migration with respect to the direction of EC alignment, and meandering index^45^, which measures directional persistence of migration, of individual T cells from the trajectories (Fig. 5B). Flow and EC alignment directions were assigned to x-axis, thus d_x_ will be close to 0.5 if T cells migrate randomly and d_x_ will be significantly greater than 0.5 if T cells crawl along the EC alignment direction. Meandering index ranges between 0 and 1, and increases as directional persistence increases. d_x_ values of T cells crawling on untreated and control ECs were significantly greater than those of T cells on LMNA-KD ECs (Fig. 5C), meaning EC A-type lamins play a certain role in crawling direction of T cells. Meandering index of T cells were not affected by the EC A-type lamins (Fig. 5D). When closely examined, most of T cells on control or untreated ECs avoided crossing over the EC nuclei (Movie S2 in SD), as reported previously^28^. To quantitatively assess the behavior of T cells encountering EC nuclei, we defined the probability of T cells crossing over the EC nuclei (P_n_) by the ratio of total number of T cells crossing over EC nuclei (N_c_) and total number of T cells encountering EC nuclei (N_e_) (left panel of Fig. 5E). Distorted nuclei of LMNA-KD ECs (Fig. 2C) may present shorter route for T cells to cross over than ellipse-shaped nuclei of control or untreated ECs do. In such cases, T cells may cross over the nuclei of LMNA-KD ECs without recognizing them, and as a result, P_n_ values would be significantly reduced. To minimize this potential artifact, we only considered T cells encountering ECs whose minimum length of nuclei were greater than 10 μm, a typical dimension of T cells^46^,^47^, so that significant portions of T cells will be on EC nuclei during their crossing over the EC nuclei. Clearly, P_n_ values of T cells crawling on LMNA-KD ECs were significantly higher than those of T cells crawling on control or untreated ECs (right panel of Fig. 5E). This result indicates that T cells sense mechanical properties of nuclei of underlying ECs and steer their leading edge protrusion against stiff nuclei regions of ECs.

**Figure 6:**
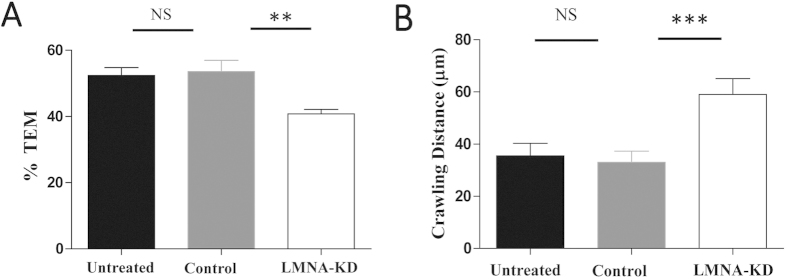
Effect of EC A-type lamins on TEM of T cells. (**A**) Percentage of T cells that successfully underwent TEM among crawling T cells. Untreated ECs, *n* = 234; Control ECs, *n* = 230; LMNA-KD ECs, *n* = 279 in total. (**B**) Crawling distances of T cells before they underwent TEM. n = 50 for each condition. Data are representative of 6 independent experiments (Mann-Whitney U-test, two-tailed, NS: not significant, **p < 0.01, ***p < 0.001).

EC A-type lamin expression levels also affected TEM of T cells. Significantly lower fractions of T cells on LMNA-KD ECs underwent TEM than T cells on control and untreated ECs (Fig. 6A). It is possible that EC A-type lamins may directly regulate TEM of T cells. It is also possible that altered migration patterns of T cells on LMNA-KD ECs prevented T cells from finding optimal route to the sites for TEM, resulting in more detachment and less TEM of T cells during crawling^28^. To test this possibility, we measured distances of crawling (Fig. 6B) before T cells undergo TEM. T cells on LMNA-KD ECs crawled much longer distances to find sites for TEM than T cells on control or untreated ECs, suggesting that reduced frequencies of TEM of T cells on LMNA-KD ECs are mostly due to altered motility patterns.

SiRNA treatment in general may alter expression of genes other than target genes by unexpected off-target effects. To rule out the possibility that our key results are affected by off-target effects of the siRNA used for the bulk of the experiments, we synthesized two siRNAs targeting different sites of LMNA, and assessed their effects on EC nuclear shape and T cell dynamics on EC layers. Regardless of siRNA sequences, LMNA-KD ECs exhibited distorted nucleus with significantly reduced circularity (Fig. S4A in SI). In addition, P_n_ values of T cells were significantly increased (Fig. S4B in SI) and the percentage of T cells successfully performing TEM was significantly reduced (Fig. S4C in SI) if ECs were treated with any of three siRNAs.

### Roles of endothelial A-type lamins in subendothelial migration of T cells

**Figure 7:**
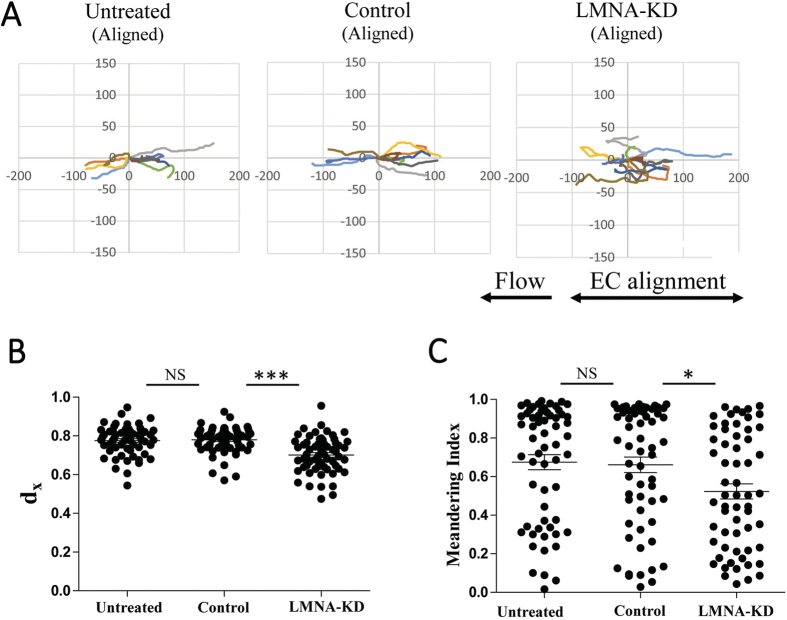
Effect of EC A-type lamins on subendothelial migration of T cells. (**A**) Representative trajectories of T cells undergoing subendothelial migration under the untreated, control and LMNA-KD ECs. (**B**,**C**) Effect of A-type lamins on d_x_ values (**B**) and meandering indexes (**C**) of T cell subendothelial migration under the untreated, control and LMNA-KD. Data are representative of 6 independent experiments (n = 60 for each condition) (Mann-Whitney U-test, two-tailed, NS: not significant, *p < 0.01, ***p < 0.001).

Leukocytes that have successfully undergone TEM by breaching endothelial layers migrate substantial distances under endothelium to breach basement membrane and enter into interstitial spaces^22^,^29^,^48^. Considering that T cells in abluminal spaces formed between endothelium and networks of pericytes/basement membrane are tightly confined, stiff nuclei of cells comprising abluminal spaces may play certain roles in the subendothelial migration of T cells. In our experimental settings, T cells undergone TEM rapidly migrated in spaces formed between ventral surfaces of inflamed EC monolayers and nanogrooved surfaces (Fig. 4B). Consistent with the previous observation^24^, when lymphocyte function-associated antigen-1 (LFA-1), a T cell integrin interacting with ICAM-1 on ECs, was blocked by adding high concentration of antibody, T cells under EC layers were no longer motile (Movie S4 in SD). This result indicates that ICAM-1 expressed on subluminal regions of ECs^24^,^49^ is more responsible for subendothelial migration of T cells than extracellular matrix molecules deposited on nanogrooved surfaces. Similar to the case with T cell crawling on EC layers, subendothelial migration of T cells was quantitatively analyzed by tracking T cells in DIC images acquired during parallel flow chamber assays. As demonstrated above (Fig. 4B), T cells on EC layers and under EC layers can be clearly distinguished by the presence of dark shadows surrounding T cells in DIC images. Trajectories of individual T cells undergoing subendothelial migration were plotted (Fig. 7A), and d_x_ and meandering index were calculated from the trajectories (Fig. 7B,C, respectively). T cells under the untreated and control ECs migrated along the EC alignment directions with almost straight trajectories. In contrast, T cells under LMNA-KD ECs frequently changed directions and exhibited substantial displacements perpendicular to the EC orientation compared to T cells under untreated or control ECs. Quantitatively, average d_x_ value and meandering index of T cells under LMNA-KD ECs were significantly lower than those of T cells under untreated or control ECs, confirming the observations. These results suggest that stiffness of EC nucleus plays an important role in the subendothelial migration of T cells as well as intraluminal crawling. However, in case of T cell migration on EC layers, meandering index of T cells on LMNA-KD ECs were comparable to that of T cells on untreated or control ECs (Fig. 5D), meaning that the directional persistence of T cell migration was significantly altered by EC nuclear stiffness only for T cells under ECs, not for T cells on ECs.

**Figure 8:**
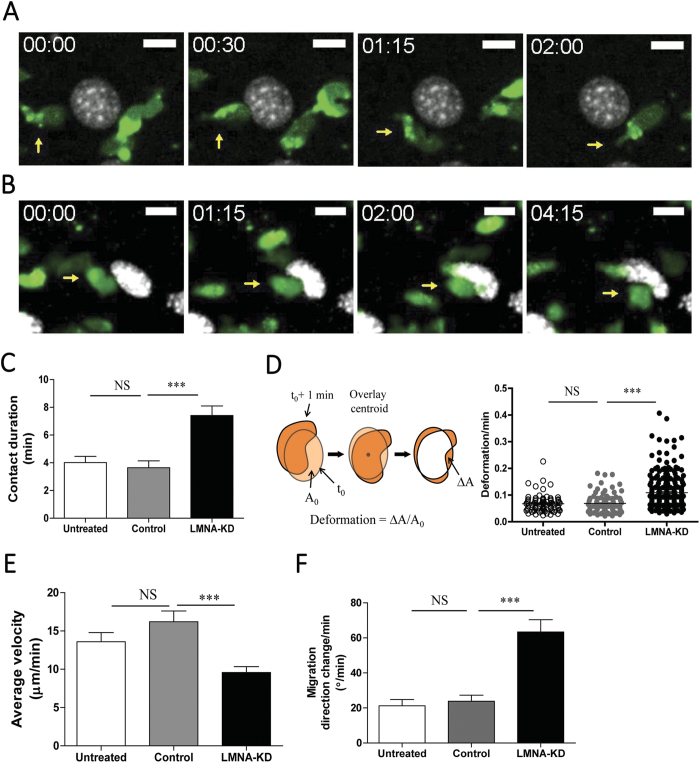
Quantitative analysis of interactions between T cells and EC nuclei during subendothelial migration of T cells. (**A**,**B**) Representative time-lapse images of T cells (labeled with CFSE, green) interacting with control (**A**) and LMNA-KD (**B**) EC nuclei (stained with Hoechst, gray). Scale bar: 10 μm. Elapse time: mm:ss. (**C**) Contact duration of EC nuclei and T cells during subendothelial migration. (**D**) Definition of nuclei deformation and nuclear deformation/min of untreated, control and LMNA-KD ECs. (**E**) Average velocity of T cells interacting with EC nuclei during subendothelial migration. (**F**) Migration direction change/min of T cells under the untreated, control and LMNA-KD ECs. Untreated ECs, *n* = 25; Control ECs, *n* = 26; LMNA-KD ECs, *n* = 30 in total. Data are representative of 4 independent experiments (Mann-Whitney U-test, two-tailed, NS: not significant, **p < 0.01, ***p < 0.001).

To better understand interactions between T cells and EC nuclei during subendothelial migration, T cells and EC nuclei were labeled with CellTrace^TM^ CFSE (or Vybrant DyeCycle^TM^) and Hoechst 33342, respectively, and time-lapse fluorescence microscopy was performed at 20 s of intervals. The majority of T cells under untreated or control ECs smoothly migrated around the EC nuclei with minimally deforming them. (Fig. 8A and Movie S5 in SI). In sharp contrast, T cells under LMNA-KD ECs frequently pushed and deformed EC nuclei and roamed around EC nuclei with significant changes in directions (Fig. 8B and Movie S6 in SI). Distinct interactions between EC nuclei-T cells under LMNA-KD ECs were quantitatively assessed by measuring contact duration, EC nuclear deformation/time, average velocity, and migration direction change/time of T cells (Fig. 8C–F). First, initiation and termination of contacts between a T cell and EC nucleus were determined by carefully examining overlaid time-lapse images (e.g. Fig. 8A,B). Then, average instantaneous velocity of T cells during contacts were measured. To measure EC nuclear deformation/time and migration direction change/time, two consecutive images acquired by time-lapse imaging (with 20 s intervals) were compared. LMNA-KD EC nuclei made significantly longer contact with T cells than untreated or control EC nuclei did (Fig. 8C). In addition, LMNA-KD ECs exhibited ~1.3-fold more deformation of nuclei/min than did untreated or control ECs (Fig. 8D). Average velocity of T cells under LMNA-KD ECs was significantly lower than that of T cells under untreated and control ECs (Fig. 8E). Finally, migration direction change of T cells/min under LMNA-KD ECs was ~3-fold higher than that of T cells under untreated or control ECs (Fig. 8F). Taken together, stiff EC nuclei supported rapid subendothelial migration of T cells with directional persistence by allowing minimal interactions between T cells and EC nuclei with minimal contact duration and direction changes.

## Discussion

Nuclear stiffness mediated by A-type lamin expression in migrating cells has shown to be a critical factor for their migration in confined acellular microenvironments^14^,^15^,^17^,^50^,^51^, but how nuclear stiffness of cells comprising microenvironments affects cell migration has not been investigated. Here, we demonstrated that T cell migration on and under EC layers were significantly influenced by the EC nuclear stiffness, suggesting that stiff nuclei of cells comprising cell-dense microenvironments can be critical mechanical cues guiding migration of other cells.

First, we provide evidences that stiff EC nuclei can serve as a “duro-repulsive” cue for intraluminal crawling of T cells. As previously reported by our group, T cells crawling on inflamed EC layers avoided crossing over EC nuclei, and cdc42-mediated invasive filopodia generation under shear^24^ was critical for such avoidance^28^. In this study, we further demonstrated that frequencies of avoiding crossing over EC nuclei were significantly decreased when EC A-type lamin expression levels were reduced by siRNA-mediated LMNA-KD. These results suggest that invasive filopodia generated by T cells is directly used to sense mechanical properties of underlying ECs to steer leading edge protrusion toward less stiff regions of EC layers. Indeed, Carman group recently reported that T cells actively probed mechanical properties of underlying endothelial layers to find regions where mechanical properties are weakest to finally perform TEM with minimal resistance (tenertaxis)^27^. Importantly, TEM never occur through EC nuclei^25^, which is typically an order of magnitude stiffer than cytoplasm^11^,^12^,^13^. Therefore, duro-repulsive behaviors with respect to stiff EC nuclei observed in the current study would be one of the efficient strategies for ‘tenertaxis’ to find sites with minimal resistance by excluding large portion of unnecessary areas and focus on more feasible regions.

These results extend current views of how mechanical microenvironments regulate cell migration. Stiffness of substrates underlying cells has been shown to be important for adhesion and migration of cells^52^. Durotaxis, cell migration directed by the gradient of substrate stiffness mostly mediated by variation in crosslinking density of acellular substrates, has been extensively studied over last decade^53^,^54^. Here, we demonstrated that cell layers composed of two mechanically distinct regions, a region containing nucleus and the other region solely containing cytoplasm, can also direct cell migration in a “duro-repulsive” manner. While pulling forces mediated by focal adhesion complexes formed in strongly adherent cells such as fibroblasts and epithelial cells are a major factor probing substrate stiffness for durotaxis^55^,^56^, direct indention by shear stress-mediated invasive filopodia^24^ appeared to be a major player sensing stiff nuclei that are located underneath thin cytoplasm.

Second, we demonstrated that stiff EC nuclei promote fast and directionally persistent migration of T cells underneath ECs. T cells encountering EC nuclei smoothly migrated around them with minimally altered motility (Fig. 8A). In contrast, T cells encountering LMNA-KD EC nuclei slowed down, made prolonged contact and substantially deformed EC nuclei, and eventually left with significantly altered motility (Fig. 8B–F). These results suggest that EC nuclear stiffness is a critical factor determining efficient migration of T cells underneath by regulating T cell-EC nucleus interactions. Stiff EC nuclei appeared to support minimal interactions between T cells and EC nuclei by readily bouncing colliding T cells, resulting in minimal changes in migration speed and direction of T cells by EC nuclei. Decreased EC nucleus stiffness by reduced expression of A-type lamins caused prolonged interactions between T cells and EC nuclei with substantial energy loss in T cells for unnecessarily deformation of EC nuclei, resulting in significant changes in migration speed and direction. Therefore, EC nuclear stiffness mediated by A-type lamins is critical for the efficient subendothelial migration of T cells.

A-type lamin-mediated nuclear stiffness has been mostly considered to be a limiting factor for cell migration in confined spaces composed of acellular structures such as collagen networks or porous membranes^1^,^14^,^15^,^17^. In contrast to this negative aspect, in this study, we provided evidence that A-type lamin-mediated nuclear stiffness of cells in cell-dense microenvironments can support migration of cells within such microenvironments by allowing efficient interactions between migrating cells and the nuclei of surrounding cells. Whether this finding can be extended to other cell-dense microenvironments such as lymph nodes, fat tissues, and tumor masses^57^,^58^,^59^ would be an interesting topic for future study.

Considering impeded migration of T cells was caused by reduced expression of EC A-type lamins, defects in EC A-type lamins may result in immunosuppression by delaying leukocyte tissue infiltration. Major disease phenotypes of LMNA mutant patients or LMNA knock-out mice reported up to date are mostly related with skeletal or cardiac muscles where mechanical force is constantly applied^1^,^60^,^61^. As far as we know, immunosuppression has not been reported in laminopathies, potentially too mild to be recognized compared with other severe symptoms.

Abluminal spaces of native endothelium are composed of pericytes and basement membrane, and their roles in directing leukocyte migration have been investigated recently^22^,^62^,^63^. Compared with native endothelium *in vivo*, abluminal spaces in our *in vitro* model system are relatively simple, but such simplicity allowed us to independently investigate the role of EC nuclear stiffness on subendothelial migration of T cells. While physiological relevance of our subendothelial migration study needs to be determined, our findings may have more general implications for cell migration in cell-dense microenvironments where migrating cells will constantly interact with the nuclei of the surrounding cells.

In summary, roles of endothelial nuclear stiffness in T cell migration on and under EC layers were investigated by quantitatively comparing motility of T cells on and under untreated, control and LMNA-KD EC layers. We found evidences that endothelial nuclear stiffness served as distinct biomechanical cues facilitating T cell motility near inflamed endothelium to enhance extravasation of T cells. This result will provide new insights into the cell migration in complex microenvironments.

## Methods

### Nanostructured surface fabrication

Nanostructured surfaces used to align EC monolayers were fabricated as previously described^28^,^34^. Briefly, nanoscale ridge/groove structures (700 nm ridges, 350 nm grooves and 300 nm heights) on a poly(ethylene terephthalate) (PET) film were replicated to a glass coverslip with 18 mm diameter (Marienfeld) by UV-assisted capillary force lithography (CFL)^64^. A UV-curable resin poly(urethane acrylate) (PUA) (Minuta Tech. Korea) was used for the replication.

### Cell culture

bEnd.3 cells (ATTC) were grown in DMEM medium (Invitrogen) supplemented with 10% FBS (Gibco) and 1% penicillin-streptomycin (PS, Invitrogen). To obtain well-aligned EC monolayers, nanostructured surfaces treated with air plasma (200~500 w, Femto Science, Korea) for 30 s were dipped into 0.1% of gelatin (Sigma) in DI water for 30 min at 37 °C and rinsed with PBS. Then 1 ml of 5 × 10^4^ cells/ml bEnd.3 cells in DMEM medium were added onto the gelatin-coated nanostructured surfaces in a well of 12-well plate and a confluent monolayer of bEnd.3 cells was used in experiments at the 4^th^ day of seeding. DO11.10 T blasts were prepared from cells in spleens and lymph nodes of DO11.10 T cell receptor transgenic mice (Jackson Laboratories) bred in the POSTECH Biotech Center (PBC). All the T cell assay including mice treatments were approved by the Institutional Animal Care and Use Committee at PBC and performed in accordance with the approved guidelines. Cells isolated from spleens and lymph nodes of DO11.10 mice were stimulated with 1 μg/ml of OVA323–339 peptide (ISQAVHAAHAEINEAGR, Peptron, Inc. Korea) and cultured in RPMI 1640 (Invitrogen) supplemented with 10% FBS, 1% PS (Invitrogen), and 0.1% beta-mercaptoethanol (Sigma). 1–2 U/ml of IL-2 (Peprotech) was added on the 2^nd^ day of OVA323–339 peptide stimulation and all experiments were performed with cells from the 5^th^ days.

### siRNA treatment

All siRNAs (LMNA siRNA-1, sense: 5′-GAGAUCGAUAACGGGAAGC(dTdT)-3′ and antisense: 5′-GCUUCCCGUUAUCGAUCUC(dTdT)-3′; LMNA siRNA-2, sense: 5′-CCCA-CCGAAGUUCACCCUAAA(dTdT)-3′ and antisense: 5′-UUUAGGGUGAACUUCGGU-GGG(dTdT)-3′; LMNA siRNA-3, sense: 5′-GGUGGUGACGAUCUGGGCU(dTdT)-3′ and antisense: 5′-AGCCCAGAUCGUCACCACC(dTdT)-3′; and control siRNA, sense: 5′-CCU-ACGCCACCAAUUUCG(dTdT)-3′ and antisense: 5′-ACGAAAUUGGUGGCGUAGG-(dTdT)-3′) were purchased from Bioneer (Korea). For siRNA treatment, culture medium of bEnd.3 cells was replaced with 1.2 ml of FBS- and PS-free DMEM medium containing 30 nM of siRNA, 0.1% of Lipofectamin RNAiMAX (Invitrogen) and 17% of Opti-MEM (Gibco) after 18 h of cell culture. After 24 h of treatment, the siRNA-containing medium was exchanged to DMEM medium containing 10% of FBS, and cultured another 24 h to obtain monolayers of bEnd.3 cells.

### Western blot

bEnd.3 cells on glass surfaces were lysed with ice-cold radioimmunoprecipitation assay (RIPA) buffer containing protease inhibitor cocktail (Abcam) and scraped by a cell scraper. Protein samples in 30 μl of Laemmli sample buffer (4% SDS, 10% 2-mercaptoehtanol, 20% glycerol, 0.004% bromophenol blue and 0.125 M Tris in DI water) were run on the wells of 12% SDS-PAGE gel and transferred to polyvinylidene fluoride (PVDF) membranes (Pierce). Then, PVDF membranes were incubated in blocking buffer (5% of bovine serum albumin (BSA) and 0.1% of Tween-20 in TBS buffer) for 1 h at room temperature, and treated with primary antibodies (anti-Lamin A/C and anti-β-actin, Abcam) and horseradish peroxidase-conjugated secondary antibodies (Pierce). The signal was revealed on CL-XPosure Film by autoradiography with enhanced chemiluminescent substrate (ECL) (Pierce) and Image J (NIH) was used for quantification of the average grey value of bands.

### Flow Cytometry

Flow cytometry was used to evaluate expression levels of A-type lamin in ECs. For staining inner nuclear membrane protein, cells were fixed and permeabilized with Foxp3 Fixation/Permeabilization working solution according to the manufacturer’s protocol (eBioscience). For staining, anti-lamin A/C (abcam) was used as a primary antibody and FITC conjugated anti-rabbit IgG (Jackson Immuno Research) was used as a seconday antibody. Antibody staining and washing were performed using in Permeabilization Buffer (eBioscience). LSR Fortessa (BD Bioscience) was used for flow cytometry, and data was analyzed using FlowJo.

### Fluorescence microscope

A modified Zeiss Axio Observer.Z1 epi-fluorescence microscope with 20× (Plan-Neofluar, NA = 0.5) and 40× (Plan-Neofluar, NA = 1.3) objective lenses and a Roper Scientific CoolSnap HQ2 CCD camera were used for imaging. XBO 75 W/2 Xenon lamp (75 W, Osram) and DAPI (EX. 365, BS 395, EMBP445/50), eGFP (EX BP 470/40, BS 495, EMBP 525/50), Cy3 (EX BP 550/25, BS 570, EMBP 605/70), Cy5 (EX BP 620/60, BS 660, EMBP 770/75) filter sets were used for fluorescence imaging. The microscope was automatically controlled by Axiovision 4.6 (Carl Zeiss); acquired images were analyzed and processed with Methamorph (Universal Imaging, Molecular Devices) or Image J.

### Confocal imaging

To analyze distribution of cytoskeletons in ECs and topography of EC layers, fluorescence imaging of nuclei, F-actin, and microtubules was performed using a confocal microscope (Carl Zeiss LSM 700). For staining, DAPI (Lifetechnology), Alexa Fluor® 647-phallodin (Lifetechnology) and anti-β-Tubulin−Cy3 antibody (Sigma) were used. Fluorescence images of stained ECs were acquired by optical z-sectioning (0.5 μm apart) using a 40× objective lens. 3D reconstructed and projected fluorescence images along the z-axis were obtained using Image J.

### >Parallel plate chamber assay

A confluent monolayer of bEnd.3 cells on nanostructured surfaces was treated with TNF-α (10 ng/ml) for 4 h and SDF-1α (100 ng/ml, PeproTech) for 10 min in order. Then, the activated bEnd.3 cell monolayer was mounted in a shear chamber (Chamlide CF, Live Cell Instrument, Korea) and the direction of EC alignment was adjusted parallel to the flow direction (or parallel to the channel longitudinal direction; 0.2 mm height, 2 mm width and 17 mm length of microchannel). DO11.10 T cells suspended in culture medium with a concentration of 2 × 10^6^ cells/ml were perfused over the bEnd.3 cell monolayer through the inlet of shear chamber using a syringe pump (New Era Pump Systems, US). Inline and stage heaters (Live Cell Instrument, Korea) were used to maintain constant temperature (37 °C). For the first 10 min, DO11.10 blasts suspended in culture medium were perfused at shear stress 0.25 dyne/cm^2^ to accumulate DO11.10 blasts on the bEnd.3 cell monolayer. Then, the blank culture medium was applied at shear stress 2 dyne/cm^2^. Subsequently, time-lapse images were acquired with 15 s interval for 20 min.

### Live cell fluorescence imaging of T cells and nuclei of ECs

To visualize dynamics of T cells and nuclei of ECs, a confluent monolayer of bEnd.3 cells treated with TNF-α and SDF-1α were stained with Hoechst 33342 (5 μg/ml, Life Technologies) for 20 min. Then, DO11.10 T cell blasts labeled with CellTrace^TM^ CFSE (1 μM, Life Technologies) or Vybrant DyeCycle^TM^ (0.5 μM, Life Technologies) were perfused on Hoechst-labeled bEnd.3 cells and each fluorescence image was sequentially acquired by time-lapse imaging for 15 min with 20 s interval.

### Statistical analysis

Statistical significance was tested using the Mann-Whitney U-test. For bar graphs, average values with standard error of mean (s.e.m.) are presented.

## Additional Information

**How to cite this article**: Song, K. H. *et al.* Roles of endothelial A-type lamins in migration of T cells on and under endothelial layers. *Sci. Rep.* **6**, 23412; doi: 10.1038/srep23412 (2016).

## Supplementary Material

Supplementary Information

Supplementary Movie S1

Supplementary Movie S2

Supplementary Movie S3

Supplementary Movie S4

Supplementary Movie S5

Supplementary Movie S6

## Figures and Tables

**Figure 1:**
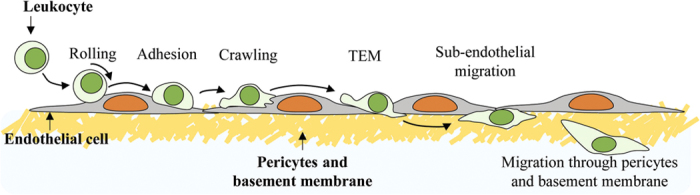
Schematic illustration of leukocyte adhesion cascade in inflamed blood vessels.

## References

[b1] Davidson P. M. & Lammerding J. Broken nuclei–lamins, nuclear mechanics, and disease. Trends. Cell Biol. 24, 247–256 (2014). 24309562 10.1016/j.tcb.2013.11.004 PMC3972295

[b2] Isermann P. & Lammerding J. Nuclear mechanics and mechanotransduction in health and disease. Curr. Biol. 23, R1113–R1121 (2013). 24355792 10.1016/j.cub.2013.11.009 PMC3883624

[b3] Devos D. P., Gräf R. & Field M. C. Evolution of the nucleus. Curr. Opin. Cell Biol. 28, 8–15 (2014). 24508984 10.1016/j.ceb.2014.01.004 PMC4071446

[b4] Ho C. Y. & Lammerding J. Lamins at a glance. J. Cell Sci. 125, 2087–2093 (2012). 22669459 10.1242/jcs.087288 PMC3367936

[b5] Ivanovska I. L., Shin J. W., Swift J. & Discher D. E. Stem cell mechanobiology: diverse lessons from bone marrow. Trends. Cell Biol. 25, 523–532 (2015). 26045259 10.1016/j.tcb.2015.04.003 PMC4555184

[b6] Shin J. W. *et al.* Lamins regulate cell trafficking and lineage maturation of adult human hematopoietic cells. P. Natl. Acad. Sci. USA 110, 18892–18897 (2013). 10.1073/pnas.1304996110 PMC3839750 24191023

[b7] Lammerding J. *et al.* Lamins A and C but not lamin B1 regulate nuclear mechanics. J. Biol. Chem. 281, 25768–25780 (2006). 16825190 10.1074/jbc.M513511200

[b8] Swift J. *et al.* Nuclear lamin-A scales with tissue stiffness and enhances matrix-directed differentiation. Science 341, 1240104 (2013). 23990565 10.1126/science.1240104 PMC3976548

[b9] Friedl P. & Alexander S. Cancer invasion and the microenvironment: plasticity and reciprocity. Cell 147, 992–1009 (2011). 22118458 10.1016/j.cell.2011.11.016

[b10] G Gritsenko, P., Ilina, O. & Friedl, P. Interstitial guidance of cancer invasion. J. Pathol. 226, 185–199 (2012). 22006671 10.1002/path.3031

[b11] Dahl K. N., Kahn S. M., Wilson K. L. & Discher D. E. The nuclear envelope lamina network has elasticity and a compressibility limit suggestive of a molecular shock absorber. J. Cell Sci. 117, 4779–4786 (2004). 15331638 10.1242/jcs.01357

[b12] Caille N., Thoumine O., Tardy Y. & Meister J.-J. Contribution of the nucleus to the mechanical properties of endothelial cells. J. Biomech. 35, 177–187 (2002). 11784536 10.1016/s0021-9290(01)00201-9

[b13] Guilak F., Tedrow J. R. & Burgkart R. Viscoelastic properties of the cell nucleus. Biochem. Bioph. Res. Co. 269, 781–786 (2000). 10.1006/bbrc.2000.2360 10720492

[b14] Rowat A. C. *et al.* Nuclear envelope composition determines the ability of neutrophil-type cells to passage through micron-scale constrictions. J. Biol. Chem. 288, 8610–8618 (2013). 23355469 10.1074/jbc.M112.441535 PMC3605679

[b15] Harada T. *et al.* Nuclear lamin stiffness is a barrier to 3D migration, but softness can limit survival. J. Cell Biol. 204, 669–682 (2014). 24567359 10.1083/jcb.201308029 PMC3941057

[b16] Wolf K. *et al.* In Seminars in cell & developmental biology. 931–941 (Elsevier, 2009).19682592

[b17] Wolf K. *et al.* Physical limits of cell migration: control by ECM space and nuclear deformation and tuning by proteolysis and traction force. J. Cell Biol. 201, 1069–1084 (2013). 23798731 10.1083/jcb.201210152 PMC3691458

[b18] Lautenschläger F. & Piel M. Microfabricated devices for cell biology: all for one and one for all. Curr. Opin. Cell Biol. 25, 116–124 (2013). 23195438 10.1016/j.ceb.2012.10.017

[b19] Jacobelli J. *et al.* Confinement-optimized three-dimensional T cell amoeboid motility is modulated via myosin IIA-regulated adhesions. Nat. Immunol. 11, 953–961 (2010). 20835229 10.1038/ni.1936 PMC2943564

[b20] Masopust D. & Schenkel J. M. The integration of T cell migration, differentiation and function. Nat. Rev. Immunol. 13, 309–320 (2013). 23598650 10.1038/nri3442

[b21] Von Andrian U. H. & Mackay C. R. T-Cell Function and Migration—Two Sides of the Same Coin. N. Engl. J. Med. 343, 1020–1034 (2000). 11018170 10.1056/NEJM200010053431407

[b22] Nourshargh S., Hordijk P. L. & Sixt M. Breaching multiple barriers: leukocyte motility through venular walls and the interstitium. Nat. Rev. Mol. Cell Biol. 11, 366–378 (2010). 20414258 10.1038/nrm2889

[b23] Ley K., Laudanna C., Cybulsky M. I. & Nourshargh S. Getting to the site of inflammation: the leukocyte adhesion cascade updated. Nat. Rev. Immunol. 7, 678–689 (2007). 17717539 10.1038/nri2156

[b24] Shulman Z. *et al.* Lymphocyte crawling and transendothelial migration require chemokine triggering of high-affinity LFA-1 integrin. Immunity 30, 384–396 (2009). 19268609 10.1016/j.immuni.2008.12.020 PMC2803105

[b25] Carman C. V. Mechanisms for transcellular diapedesis: probing and pathfinding byinvadosome-like protrusions’. J. Cell Sci. 122, 3025–3035 (2009). 19692589 10.1242/jcs.047522

[b26] Carman C. V. *et al.* Transcellular diapedesis is initiated by invasive podosomes. Immunity 26, 784–797 (2007). 17570692 10.1016/j.immuni.2007.04.015 PMC2094044

[b27] Martinelli R. *et al.* Probing the biomechanical contribution of the endothelium to lymphocyte migration: diapedesis by the path of least resistance. J. Cell Sci. 127, 3720–3734 (2014). 25002404 10.1242/jcs.148619 PMC4150060

[b28] Song K. H. *et al.* T cells sense biophysical cues using lamellipodia and filopodia to optimize intraluminal path finding. Integr. Biol. 6, 450 (2014). 10.1039/c4ib00021h 24599186

[b29] Proebstl D. *et al.* Pericytes support neutrophil subendothelial cell crawling and breaching of venular walls *in vivo*. J. Exp. Med. 209, 1219–1234 (2012). 22615129 10.1084/jem.20111622 PMC3371725

[b30] Elbashir S. M. *et al.* Duplexes of 21-nucleotide RNAs mediate RNA interference in cultured mammalian cells. Nature 411, 494–498 (2001). 11373684 10.1038/35078107

[b31] Goldman R. D. *et al.* Accumulation of mutant lamin A causes progressive changes in nuclear architecture in Hutchinson–Gilford progeria syndrome. P. Natl. Acad. Sci. USA 101, 8963–8968 (2004). 10.1073/pnas.0402943101 PMC428455 15184648

[b32] Gruenbaum Y., Margalit A., Goldman R. D., Shumaker D. K. & Wilson K. L. The nuclear lamina comes of age. Nat. Rev. Mol. Cell Biol. 6, 21–31 (2005). 15688064 10.1038/nrm1550

[b33] Mellad J. A., Warren D. T. & Shanahan C. M. Nesprins LINC the nucleus and cytoskeleton. Curr. Opin. Cell Biol. 23, 47–54 (2011). 21177090 10.1016/j.ceb.2010.11.006

[b34] Song K. H., Kwon K. W., Song S., Suh K. Y. & Doh J. Dynamics of T cells on endothelial layers aligned by nanostructured surfaces. Biomaterials 33, 2007–2015 (2012). 22189145 10.1016/j.biomaterials.2011.12.002

[b35] Restifo N. P., Dudley M. E. & Rosenberg S. A. Adoptive immunotherapy for cancer: harnessing the T cell response. Nat. Rev. Immunol. 12, 269–281 (2012). 22437939 10.1038/nri3191 PMC6292222

[b36] Rosenberg S. A., Restifo N. P., Yang J. C., Morgan R. A. & Dudley M. E. Adoptive cell transfer: a clinical path to effective cancer immunotherapy. Nat. Revi. Cancer 8, 299–308 (2008). 18354418 10.1038/nrc2355 PMC2553205

[b37] Steiner O. *et al.* Differential roles for endothelial ICAM-1, ICAM-2, and VCAM-1 in shear-resistant T cell arrest, polarization, and directed crawling on blood–brain barrier endothelium. J. Immunol. 185, 4846–4855 (2010). 20861356 10.4049/jimmunol.0903732

[b38] Cinamon G., Shinder V. & Alon R. Shear forces promote lymphocyte migration across vascular endothelium bearing apical chemokines. Nat. Immunol. 2, 515–522 (2001). 11376338 10.1038/88710

[b39] Arnison M., Cogswell C., Smith N., Fekete P. & Larkin K. Using the Hilbert transform for 3D visualization of differential interference contrast microscope images. J. Microsc. 199, 79–84 (2000). 10886531 10.1046/j.1365-2818.2000.00706.x

[b40] Mehta S. B. & Sheppard C. J. Partially coherent image formation in differential interference contrast (DIC) microscope. Opt. Express 16, 19462–19479 (2008). 19030033 10.1364/oe.16.019462

[b41] Shribak M. & Inoué S. Orientation-independent differential interference contrast microscopy. Appl. Opt. 45, 460–469 (2006). 16463729 10.1364/ao.45.000460

[b42] Barr V. A. & Bunnell S. C. Interference reflection microscopy. Current Protocols in Cell Biology, 4.23. 21–24.23. 19 (2009). 10.1002/0471143030.cb0423s45 PMC2824538 20013754

[b43] Verschueren H. Interference reflection microscopy in cell biology: methodology and applications. J. Cell Sci. 75, 279–301 (1985). 3900106 10.1242/jcs.75.1.279

[b44] Kwon K. W. *et al.* Nanotopography-guided migration of T cells. J. Immunol. 189, 2266–2273 (2012). 22844118 10.4049/jimmunol.1102273

[b45] Choi J.-C., Jung H.-R. & Doh J. Dynamic Modulation of Small-Sized Multicellular Clusters Using a Cell-Friendly Photoresist. ACS Appl. Mater. Interfaces 5, 12757–12763 (2013). 24256472 10.1021/am404134u

[b46] Doh J., Kim M. & Krummel M. F. Cell-laden microwells for the study of multicellularity in lymphocyte fate decisions. Biomaterials 31, 3422–3428 (2010). 20117834 10.1016/j.biomaterials.2010.01.018

[b47] Kim M. *et al.* Addressable micropatterning of multiple proteins and cells by microscope projection photolithography based on a protein friendly photoresist. Langmuir 26, 12112–12118 (2010). 20565061 10.1021/la1014253

[b48] Stolp B. *et al.* HIV-1 Nef interferes with T-lymphocyte circulation through confined environments *in vivo*. Proc. Natl. Acad. Sci. USA. 109, 18541–18546 (2012). 23093676 10.1073/pnas.1204322109 PMC3494961

[b49] Oppenheimer-Marks N., Davis L. S., Bogue D. T., Ramberg J. & Lipsky P. Differential utilization of ICAM-1 and VCAM-1 during the adhesion and transendothelial migration of human T lymphocytes. J. Immunol. 147, 2913–2921 (1991). 1717579

[b50] Friedl P., Wolf K. & Lammerding J. Nuclear mechanics during cell migration. Curr. Opin. Cell Biol. 23, 55–64 (2011). 21109415 10.1016/j.ceb.2010.10.015 PMC3073574

[b51] Lee J. S. *et al.* Nuclear lamin A/C deficiency induces defects in cell mechanics, polarization, and migration. Biophys. J. 93, 2542–2552 (2007). 17631533 10.1529/biophysj.106.102426 PMC1965451

[b52] Discher D. E., Janmey P. & Wang Y.-l. Tissue cells feel and respond to the stiffness of their substrate. Science 310, 1139–1143 (2005). 16293750 10.1126/science.1116995

[b53] Raab M. *et al.* Crawling from soft to stiff matrix polarizes the cytoskeleton and phosphoregulates myosin-II heavy chain. J. Cell Biol. 199, 669–683 (2012). 23128239 10.1083/jcb.201205056 PMC3494847

[b54] Zaari N., Rajagopalan P., Kim S. K., Engler A. J. & Wong J. Y. Photopolymerization in microfluidic gradient generators: microscale control of substrate compliance to manipulate cell response. Adv. Mater. 16, 2133–2137 (2004).

[b55] Plotnikov S. V., Pasapera A. M., Sabass B. & Waterman C. M. Force fluctuations within focal adhesions mediate ECM-rigidity sensing to guide directed cell migration. Cell 151, 1513–1527 (2012). 23260139 10.1016/j.cell.2012.11.034 PMC3821979

[b56] Geiger B., Spatz J. P. & Bershadsky A. D. Environmental sensing through focal adhesions. Nat. Rev. Mol. Cell Biol. 10, 21–33 (2009). 19197329 10.1038/nrm2593

[b57] von Andrian U. H. & Mempel T. R. Homing and cellular traffic in lymph nodes. Nat. Rev. Immunol. 3, 867–878 (2003). 14668803 10.1038/nri1222

[b58] Nishimura S. *et al.* CD8 + effector T cells contribute to macrophage recruitment and adipose tissue inflammation in obesity. Nat. Med. 15, 914–920 (2009). 19633658 10.1038/nm.1964

[b59] Friedl P. & Gilmour D. Collective cell migration in morphogenesis, regeneration and cancer. Nat. Rev. Mol. Cell Biol. 10, 445–457 (2009). 19546857 10.1038/nrm2720

[b60] Stewart C. L., Kozlov S., Fong L. G. & Young S. G. Mouse models of the laminopathies. Exp. Cell. Res. 313, 2144–2156 (2007). 17493612 10.1016/j.yexcr.2007.03.026 PMC1949387

[b61] Butin-Israeli V., Adam S. A., Goldman A. E. & Goldman R. D. Nuclear lamin functions and disease. Trends in genetics 28, 464–471 (2012). 22795640 10.1016/j.tig.2012.06.001 PMC3633455

[b62] Weninger W., Biro M. & Jain R. Leukocyte migration in the interstitial space of non-lymphoid organs. Nat. Rev. Immunol. 14, 232–246 (2014). 24603165 10.1038/nri3641

[b63] Nourshargh S. & Alon R. Leukocyte Migration into Inflamed Tissues. Immunity 41, 694–707 (2014). 25517612 10.1016/j.immuni.2014.10.008

[b64] Suh K. Y., Park M. C. & Kim P. Capillary force lithography: a versatile tool for structured biomaterials interface towards cell and tissue engineering. Adv. Funct. Mater. 19, 2699–2712 (2009).

