# Supplementary material for: Roles of endothelial A-type lamins in migration of T cells on and under endothelial layers
Source: Sci Rep. 2016 Mar 21;6:23412. doi: 10.1038/srep23412 (PMC4800500; doi:10.1038/srep23412)
Supplement: Supplementary Information [file srep23412-s1.pdf]

Roles of endothelial A-type lamins in migration of T cells on and under endothelial layers

Kwang Hoon Song,[1] Jaehyun Lee,[2] HyoungJun Park,[1] Hye Mi Kim,[3] Jeehun Park,[2] Keon Woo Kwon,[1] and Junsang Doh[1,2,]*

[1]Department of Mechanical Engineering, [2]School of Interdisciplinary Bioscience and Bioengineering (I-Bio), [3]Division of Integrative Bioscience and Biotechnology (IBB), Pohang University of Science and Technology (POSTECH) San 31, Hyoja-dong, Nam-Gu, Pohang, Gyeongbuk, 790-784, Korea.

Supplementary Information (SI)

Supplementary figures

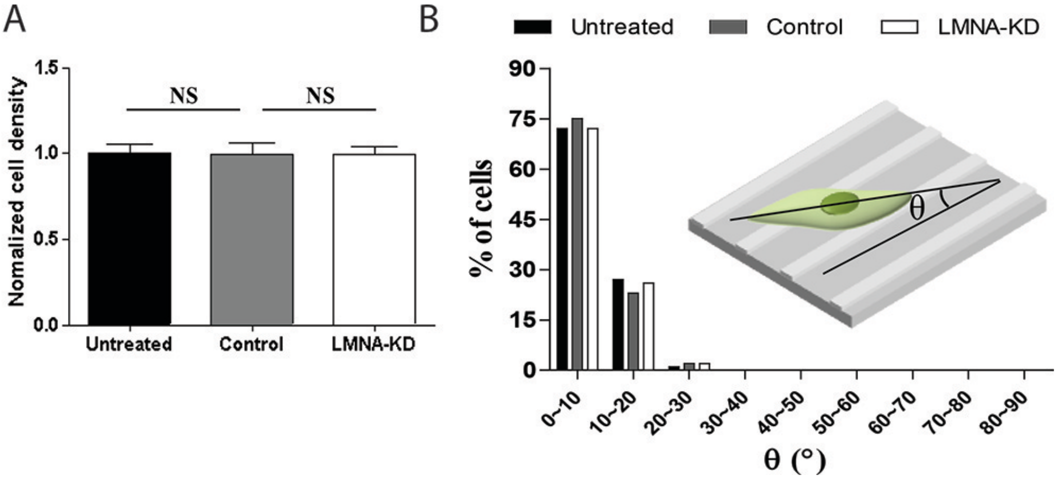

Figure S1. (A, B) Effect of reduced expression of A-type lamins on cell density (A) and cell alignment along the groove of nanostructured surfaces (B).

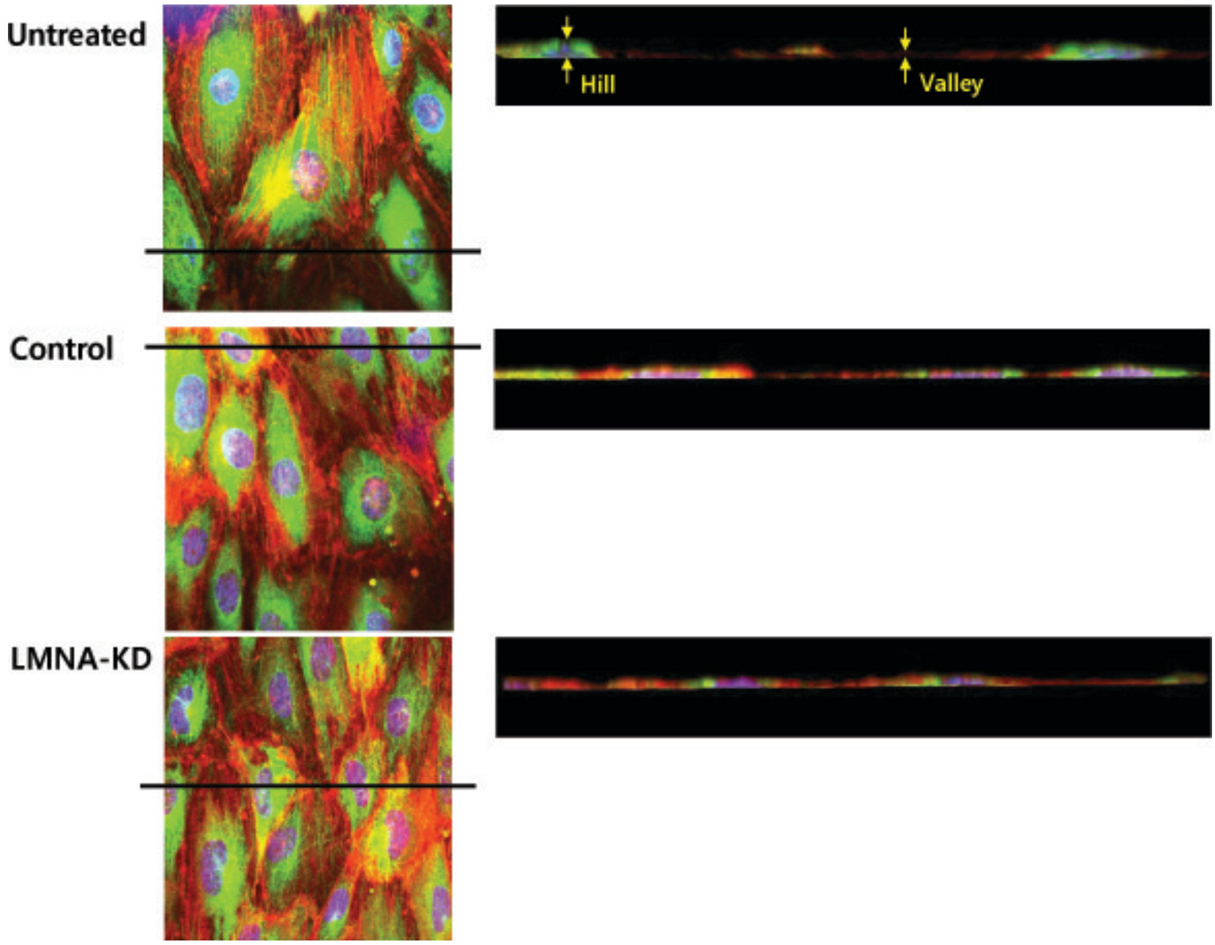

Figure S2. XY-projected (left) and cross-sectional (right) images of 3D reconstructed confocal images of EC layers stained with nucleus (blue), F-actin (red) and microtubule (green). Cross-sectional images are reconstructed from the linescans of XY-projected images. Linescans were drawn to connect centroids of two adjacent nuclei. Local maximum (typically near centroid of nuclei) and minimum were defined as 'hill' and 'valley', respectively.

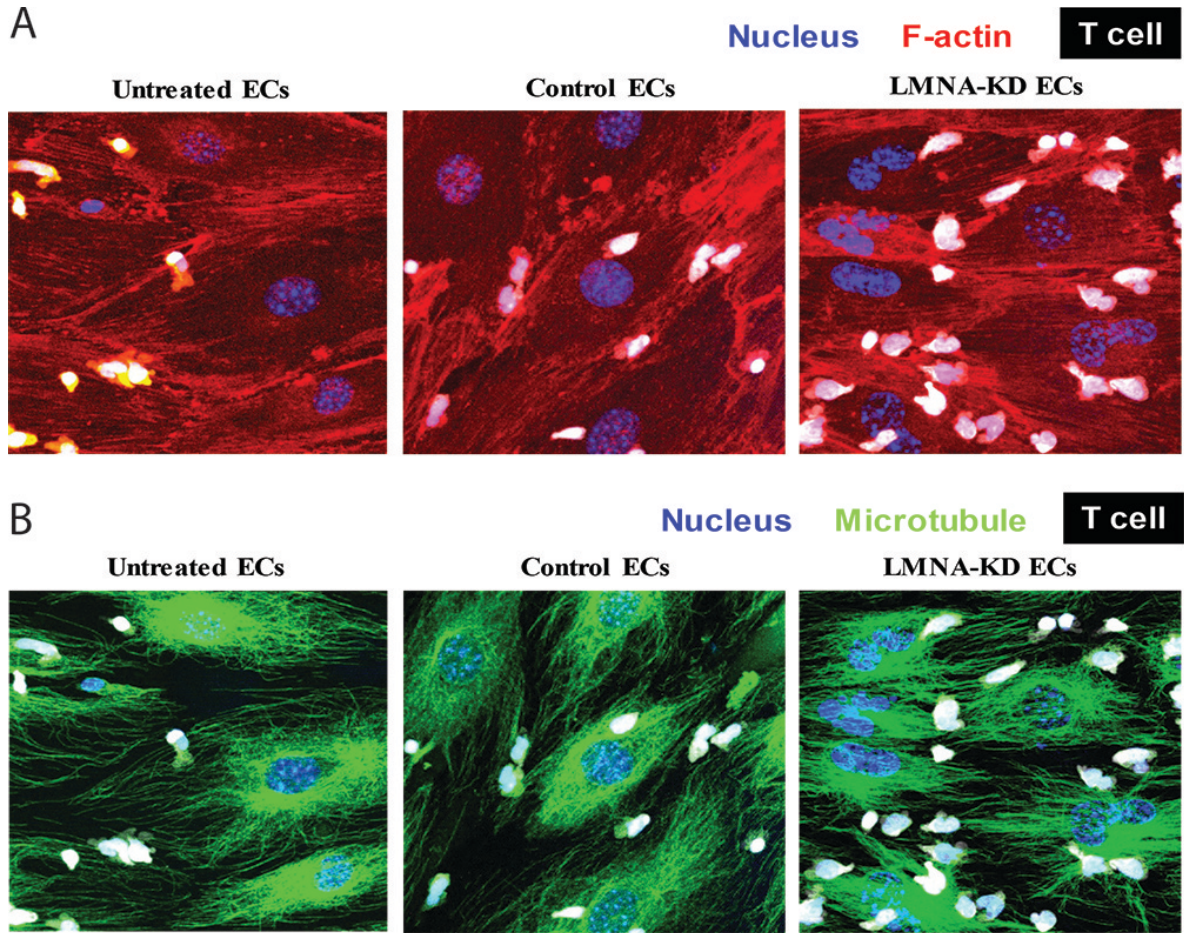

Figure S3. (A, B) Fluorescence images of F-actin (A) and microtubule (B) overlaid with nucleus (blue) and T cells (white).

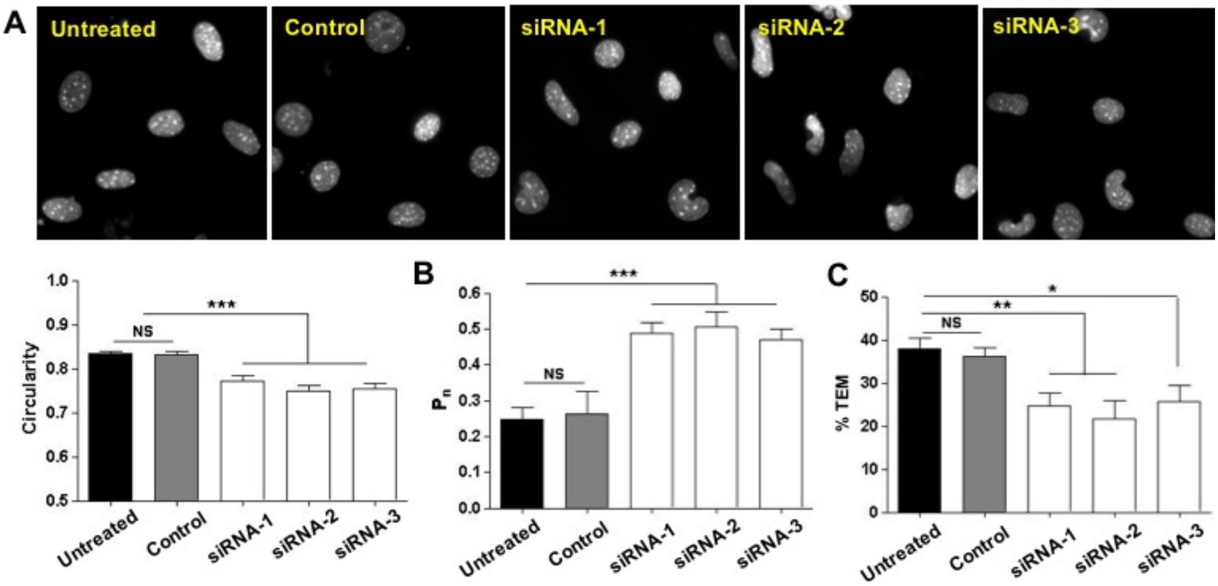

Figure S4. Effects of siRNA sequences on the EC nuclear morphology (A), directionality of T cell crawling on ECs (B), and incidence of transendothelial migration (C). Three different siRNAs, siRNA-1 (sense: 5'-GAGAUCGAUAACGGGAAGC(dTdT)-3' and antisense: 5'-GCUUCCCGUUAUCGAUCUC(dTdT)-3'), siRNA-2 (sense: 5'-CCCACCGAAGUUCA-CCCUAAA(dTdT)-3' and antisense: 5'-UUUAGGGUGAACUUCGGUGGG(dTdT)-3'), and siRNA-3 (sense: 5'-GGUGGUGACGAUCUGGGCU(dTdT)-3' and antisense: 5'-AGC-CCAGAUCGUCACCACC(dTdT)-3'), targeting different sites of murine LMNA were synthesized (Bioneer, Korea) and used. Data are representative of 3 independent experiments. (*n* > 60 for each condition) (Mann-Whitney U-test, two-tailed, NS: not significant, ***p < 0.05, **p < 0.01, ***p < 0.001).

Supplementary movie legends

Movie S1. A representative movie of a T cell undergoing transendothelial migration (TEM) acquired by two different modes of imaging, differential interference contrast (DIC, left) and interference reflection microscopy (IRM, right). Scale bar: 10 μm. Elapsed time: mm:ss.

Movie S2. A representative movie showing interactions between a T cell and control EC nucleus during intraluminal crawling. Blue: nuclei of ECs. Scale bar: 10 μm. Elapsed time: mm:ss.

Movie S3. A representative movie showing interactions between a T cell and LMNA-KD EC nucleus during intraluminal crawling. Blue: nuclei of ECs. Scale bar: 10 μm. Elapsed time: mm:ss.

Movie S4. A representative movie showing the effect of anti-LFA-1 treatment on subendothelial migrations of T cells. Scale bar: 10 μm. Elapsed time: mm:ss.

Movie S5. A representative movie showing interactions between a T cell and control EC nucleus during subendothelial migration. Green: T cells and gray: nuclei of ECs. Scale bar: 10 μm. Elapsed time: mm:ss.

Movie S6. A representative movie showing interactions between a T cell and LMNA-KD EC nucleus during subendothelial migration. Green: T cells and gray: nuclei of ECs. Scale bar: 10 μm. Elapsed time: mm:ss.
